# Chromosome numbers in three species groups of freshwater flatworms increase with increasing latitude

**DOI:** 10.1002/ece3.1969

**Published:** 2016-02-03

**Authors:** Sven Lorch, Dirk Zeuss, Roland Brandl, Martin Brändle

**Affiliations:** ^1^ Department of Ecology, Animal EcologyFaculty of BiologyPhilipps‐Universität MarburgKarl‐von‐Frisch‐Straße 835043MarburgGermany

**Keywords:** Geographical range, parthenogenesis, Platyhelminthes, polyploidy, reproduction

## Abstract

Polyploidy in combination with parthenogenesis offers advantages for plasticity and the evolution of a broad ecological tolerance of species. Therefore, a positive correlation between the level of ploidy and increasing latitude as a surrogate for environmental harshness has been suggested. Such a positive correlation is well documented for plants, but examples for animals are still rare. Species of flatworms (Platyhelminthes) are widely distributed, show a remarkably wide range of chromosome numbers, and offer therefore good model systems to study the geographical distribution of chromosome numbers. We analyzed published data on counts of chromosome numbers and geographical information of three flatworm “species” (*Phagocata vitta*,* Polycelis felina* and *Crenobia alpina*) sampled across Europe (220 populations). We used the mean chromosome number across individuals of a population as a proxy for the level of ploidy within populations, and we tested for relationships of this variable with latitude, mode of reproduction (sexual, asexual or both) and environmental variables (annual mean temperature, mean diurnal temperature range, mean precipitation and net primary production). The mean chromosome numbers of all three species increased with latitude and decreased with mean annual temperature. For two species, chromosome number also decreased with mean precipitation and net primary production. Furthermore, high chromosome numbers within species were accompanied with a loss of sexual reproduction. The variation of chromosome numbers within individuals of two of the three species increased with latitude. Our results support the hypothesis that polyploid lineages are able to cope with harsh climatic conditions at high latitudes. Furthermore, we propose that asexual reproduction in populations with high levels of polyploidization stabilizes hybridization events. Chromosomal irregularities within individuals tend to become more frequent at the extreme environments of high latitudes, presumably because of mitotic errors and downsizing of the genome.

## Introduction

Chromosome numbers and levels of polyploidy vary with latitude and elevation, a pattern well documented for plants (e.g., Löve and Löve 1943, 1967; Reese 1958; Hanelt 1966; Grant 1971; Stebbins 1971; Levin 2002; te Beest et al. 2011; Peruzzi et al. 2012). Furthermore, diploid plant species dominate in stable habitats, whereas polyploids are common in disturbed and/or early successional habitats (te Beest et al. 2011 and references therein). In contrast to the numerous studies with plants, few analyses of the relationship between environment and chromosome numbers of animals are available. These few studies have shown, for example, that polyploid individuals of the European weevil *Otiorrhynchus dubius* (Curculionidae) tolerate lower temperatures than diploid individuals (Suomalainen et al. 1987). Tetraploid populations of the bagworm moth *Solenobia triquetrella* (Psychidae) spread throughout Europe after the last glaciation, whereas diploid populations showed almost no expansion of their distributional area since the end of the last glaciation (Suomalainen et al. 1987). After glaciation events, polyploid forms of the crustacean *Pontoporeia affinis* (Amphipoda, Pontoporeiidae) expanded across a broader geographical range and diversity of habitats than the diploids (Salemaa 1984).

To explain such patterns of geographic variation in chromosome numbers or polyploidy, three main hypotheses have been suggested:

1. Polyploids harbor multiple variants of genes, which provide more opportunities for genetic recombination and advantageous mutations (Comai 2005). In consequence, polyploid populations can adapt to new environments within few generations (Stebbins 1985; Soltis and Soltis 2000; Kearney 2005; te Beest et al. 2011), which might facilitate colonization of new environments particularly at the distributional borders of a species (Bierzychudek 1985; Stebbins 1985; Soltis and Soltis 2000; Hörandl 2006; Mráz et al. 2009; te Beest et al. 2011).
2. Another hypothesis is based on the observation that at higher latitudes parthenogenetic reproduction is more common than sexual reproduction (“geographic parthenogenesis,” Vandel 1928). In addition, parthenogenetic species or populations often show higher levels of ploidy than species or populations with sexual reproduction (e.g., Bierzychudek 1985; Suomalainen et al. 1987; Turgeon and Hebert 1994; Beukeboom et al. 1998; Durka 2002; Comai 2005; D'Souza et al. 2005; Kearney 2005; Adolfsson et al. 2010; D'Souza and Michiels 2010). Although counterexamples exist (e.g., Salmonidae, Philipps and Ráb 2001; Acipenseriformes, Birstein and Vasiliev 1987), exceedingly high or uneven numbers of chromosomes tend to impair sexual reproduction (Adolfsson et al. 2010). Therefore, with an increase in the importance of parthenogenetic reproduction, the level of ploidy should also increase (Pongratz et al. 2003; Mráz et al. 2009).
3. The probability of errors during meiosis and mitosis increases with harsh environmental conditions such as severe variations in temperature, high concentrations of certain ions as well as water and nutrient stress (Ramsey and Schemske 1998). As a consequence, spontaneous polyploidization, aneuploidy, and individuals with cells differing in chromosome numbers are more common in harsh environments (Hagerup 1931; te Beest et al. 2011). This may allow some polyploids to be successful in fluctuating environments and predicts an increase of the variability of chromosome numbers *within* individuals or *among* individuals within populations with harsh and more variable conditions.

In freshwater flatworms (Platyhelminthes), chromosome numbers and levels of ploidy vary considerably across species, populations within species, individuals within populations (D'Souza et al. 2005), and even within individuals (Dahm 1958). Some Tricladida such as *Polycelis nigra* have B chromosomes and/or an extra A chromosome (aneuploidy; Beukeboom et al. 1998). Flatworms are hermaphrodites with versatile mechanisms of reproduction (Dahm 1958; Benazzi and Benazzi Lentati 1976; Reynoldson and Young 2000). Amphimixis tends to occur only in diploids or individuals with low levels of ploidy, whereas most polyploid flatworms reproduce in an apomictic manner *via* parthenogenetic pseudogamy. Thereby, the fusion of gametes is required to stimulate the development of eggs, but the male genome becomes subsequently eliminated (Dahm 1958; Benazzi and Benazzi Lentati 1976; Beukeboom et al. 1998; Pongratz et al. 2003). In addition to these two main reproductive modes, many flatworm species are able to asexually reproduce *via* fission.

Obviously, flatworms are good model species for studying the geographic variation of chromosome numbers as well as of modes of reproduction. Here, we analyzed the latitudinal variation in chromosome numbers of three species, respectively species flocks of freshwater flatworms from the family Planariidae (*Phagocata vitta*,* Polycelis felina* and *Crenobia alpina*). Specifically, we tested three predictions that follow from the above hypotheses. These predictions are based on the general assumption made in most studies of polyploidy that the environment at high latitudes calls for particular requirements that need special adaptations: Therefore, (1) chromosome numbers should increase with increasing latitude; (2) populations with asexual reproduction should be more frequent at higher latitudes; and (3) the variation of chromosome numbers within individuals should increase with increasing latitude. For our tests, we used 50‐year‐old data published by Dahm (1958). Although the marriage of old‐fashioned data and modern statistical methods may seem strange, we feel that the re‐analysis of old data provides important insights and hints for further modern investigation. Moreover, it shows that reporting and documenting descriptive data in detail provide for following generations the possibility to reanalyze the data in light of new hypotheses.

## Material and Methods

### Species and source of chromosomal data

We analyzed the extensive chromosomal data of freshwater flatworm populations published by Dahm (1958). Dahm reported, among other things, the reproduction mode and the chromosome number for individuals of five “species” of freshwater flatworms sampled across Europe: *Dugesia gonocephala* (Dugès)*, Dugesia tigrina* (Girard) (synonym *Girardia tigrina* (Girard))*, Phagocata vitta* (Dugès), *Polycelis felina* (Dalyell), and *Crenobia alpina* (Dana). These “species” probably consist of species complexes or species flocks (e.g., Dahm 1958; de Vries 1984). However, the principal ideas about the advantages of high chromosome numbers within species outlined in the introduction may also apply to the variation of chromosome numbers between higher taxonomic groups (e.g., Löve and Löve 1943; Stebbins 1971; Peruzzi et al. 2012).

In the following, we present analyses of the “species” *Ph. vitta*,* P. felina,* and *C. alpina* which represent the bulk of populations studied by Dahm (but see Discussion). Molecular and morphological analyses indicate that these “species” are closely related belonging to the family Planariidae (Sluys et al. 2009). Species of the genus *Dugesia* are assigned into a separate family (Dugesiidae; see Sluys et al. 2009). Note also that *Dugesia tigrina* was introduced into Europe from North America (e.g., Gourbault 1969). Dahm (1958) characterized the reproductive mode of each population and distinguished between sexual, asexual by fission, and populations that were able to reproduce sexually *and* asexually. Successful sexual reproduction within a population was noted if fertile cocoons were produced. Furthermore, he documented the number of chromosomes from cells of several individuals within each of 220 populations (1–18 cells per individual). Sometimes, however, the exact chromosome number could not be determined. In such cases, approximate counts were used. The chromosome numbers also differed in several individuals among cells. Therefore, we first calculated the mean value for each individual and subsequently the mean chromosome number across individuals of each population for further statistical tests.

To analyze the variation in chromosome numbers, we used two approaches. First, we calculated the coefficient of variation (CV) in chromosome number within individuals and then we calculated the mean CV across all individuals within a population. Here, we considered only those individuals where chromosome counts from more than one cell were available (606 of 1177 individuals). This reduced the total number of analyzed populations from 220 to 203. Second, we analyzed the CV among individuals within populations based on the mean number of chromosomes of each individual. Here, only those populations were considered where chromosome numbers for at least two individuals were available (205 populations).

Chromosome numbers are the most straightforward metric to characterize the karyotype. One can use such counts without knowing the basic haploid complement of the chromosomes. Nevertheless, for illustrative reasons, we also report levels of polyploidy. We estimated the haploid complement for the three species (*Ph. vitta*: seven, *P. felina*: nine, *C. alpina*: seven) by the smallest number of chromosomes that was distinguishable during repeated meiosis. Table 1 summarizes the number of populations and individuals analyzed of each species with the respective reproduction modes and ploidy levels.

**Table 1:** Overview of the sampled populations of the three species of Platyhelminthes with the respective reproduction modes and ploidy levels. Numbers in brackets refer to the number of populations/individuals considered for the analyses of the variation of chromosome number *within* individuals by averaging the coefficient of variation calculated across cell counts from single individuals (CV; for further details, see text). Errors indicate ± SD

Species	Number of			Number of populations with reproduction mode				Ploidy level		
	Populations	Individuals	Cells per individual	Asexual	Sexual	Both	Unknown	Min	Max	Mean
*Ph. vitta*	47 (43)	346 (196)	2.3 ± 1.8	32	12	2	1	3	10	5.9 ± 1.4
*P. felina*	61 (58)	299 (210)	3.6 ± 3.2	44	5	8	4	2	3	2.6 ± 0.4
*C. alpina*	112 (102)	532 (328)	2.5 ± 1.9	75	10	23	4	4	9	6.1 ± 1.3

**Figure 1:**
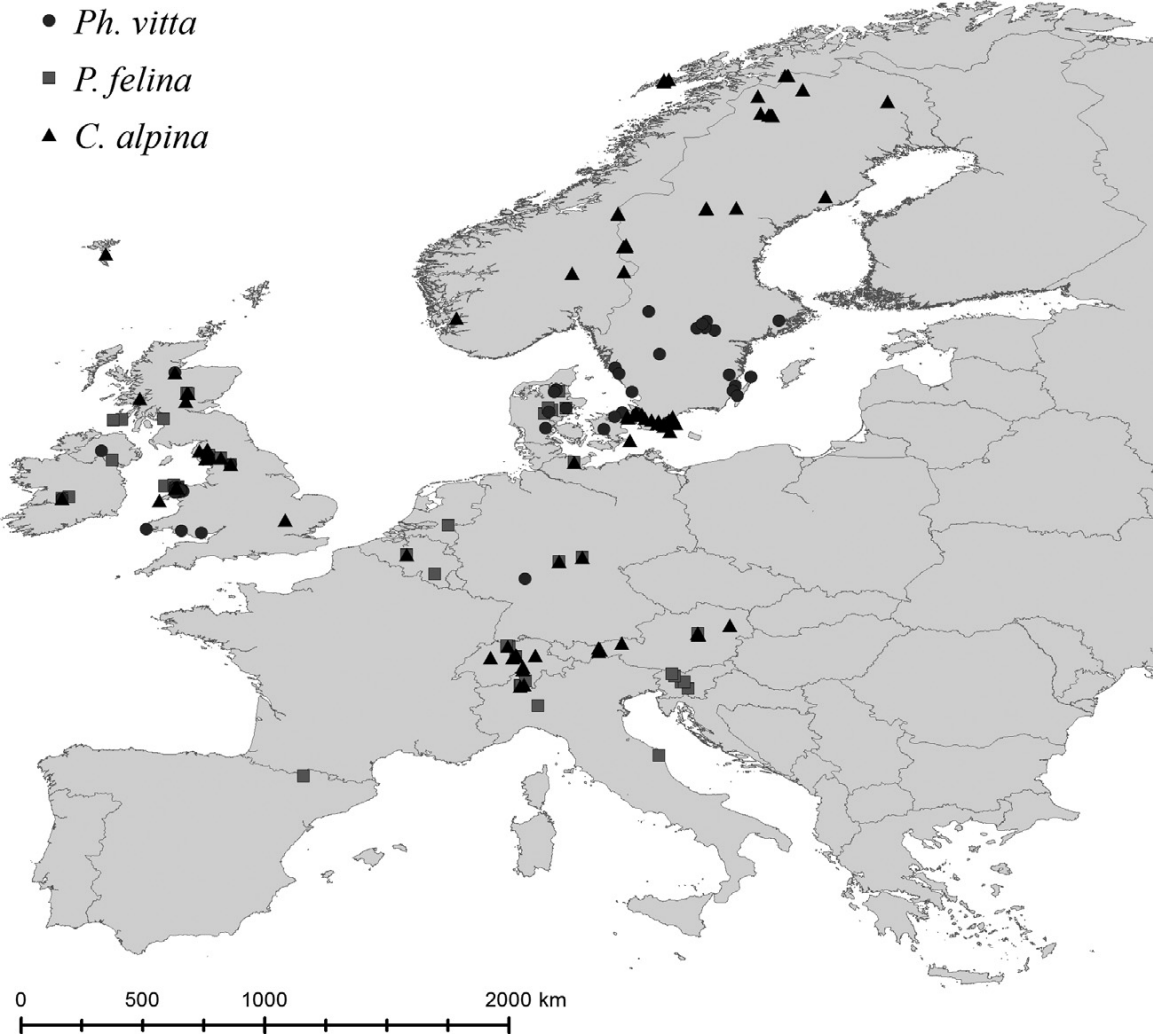
Geographic location of sampling sites of the three species of Platyhelminthes used in our study. The map was produced with Google Earth (Google Inc., 2011) and ArcMap 9.3 (ESRI, 2008).

From Dahm's descriptions of the sampling sites, we estimated latitude and longitude (Fig. 1). Dahm did not provide exact information on the geographic location of sites, but he described the geographic settings of the sampling points (e.g., country, region, nearest town, lake, mountain), which may slightly deviate from the actual locality. Because of the large latitudinal range of our study, such inaccuracies should be of minor importance. However, the available data set does not allow the analyses of chromosome numbers and reproduction mode with elevation because elevation can change within short geographic distances.

For these sampling sites, we extracted the following environmental variables from public resources using GRASS GIS (GRASS Development Team, 2012): annual mean temperature (temp), mean diurnal temperature range (temp range), annual precipitation (prec), and net primary production of biomass (NPP). These variables describe the most important environmental factors influencing ecosystems. Temperature and precipitation data were from BioClim (Hijmans et al. 2005; www.worldclim.org/bioclim, 30 arc‐seconds resolution), and NPP data were from FAO (http://www.fao.org/nr/climpag/globgrids/NPP_en.asp, 0.5 degree resolution, period 1976‐2000).

All environmental variables were negatively correlated with latitude (see Appendix S1 in Supporting Information). Therefore, we computed a principal component analysis (PCA) based on the correlation matrix of all environmental variables and latitude. PC1 and PC2 accounted for 79% of the variation (PC1 eigenvalue >2.5, 55%; PC2 eigenvalue >1, 24%). Latitude, temperature, and NPP loaded on PC1, while PC2 summarized the effect of temperature range (see Appendix S2). As latitude strongly loaded on PC1, we considered in the multiple analyses only latitude as a surrogate for PC1. A more complex multiple linear model on the effects of PC1 and PC2 and interactions with “species” and “reproduction mode” on chromosome numbers is given in the Appendix (see Appendix S3).

### Statistical analyses

After descriptive analyses, we tested for univariate relationships between the mean chromosome number and the two measures of CV in chromosome numbers of each population with latitude and for relationships between the mean chromosome number and the four environmental variables using simple linear models. For revealing possible interactions between all variables, we also applied multiple linear models. All statistical analyses were performed with R (R Development Core Team, 2013) and the package “*car*” (Fox and Weisberg 2011). The data used for the analyses are available in the Appendix (Table S1–S3).

## Results

**Figure 2:**
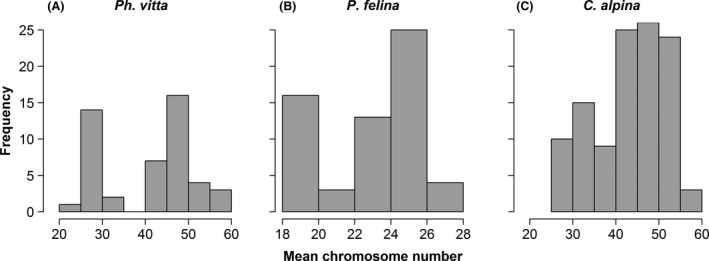
Frequency distribution of the mean chromosome numbers of populations of the examined species of Platyhelminthes (*Ph. vitta*:* n* = 47, *P. felina*:* n* = 61, *C. alpina*:* n* = 112).

**Figure 3:**
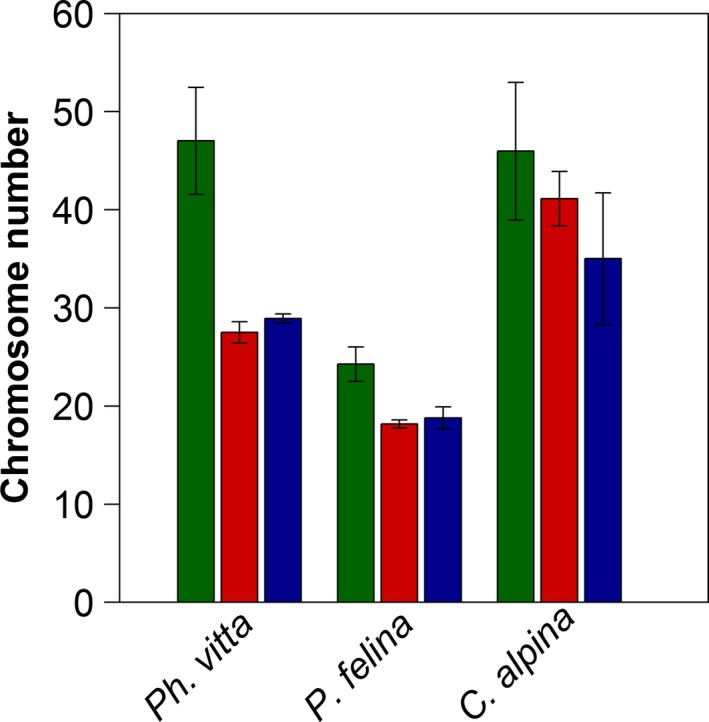
Mean chromosome numbers of populations differing in reproduction modes: green = asexual, red = sexual, blue = sexual *and* asexual. Bars represent means; error bars show ± SD (*Ph. vitta*:* n* = 46, *P. felina*:* n* = 57, *C. alpina*:* n* = 108). For nine of 220 populations, the reproduction mode was unknown.

Mean chromosome numbers of 1177 individuals across 220 populations were analyzed. *Ph. vitta* (*n* = 47 populations) showed the greatest variation in chromosome numbers among populations (range: 24.5–57.8, mean: 40.8, SD: ±10.2; Fig. 2A). The ploidy level ranged from three to ten; ploidy levels of four and six were the most frequent. The *P. felina* populations (*n* = 61 populations) showed on average lower chromosome numbers than the other two species (range: 18–27, mean: 22.8, SD: ±2.95; Fig. 2B). Many individuals of *P. felina* were diploid, but 24 to 26 chromosomes, and thus aneuploidy, were also common. The patterns in chromosome numbers of *C. alpina* populations (*n* = 112 populations) were similar to those of *Ph. vitta* (range: 28–56.4, mean: 43.2, SD: ±7.89; Fig. 2C). The smallest level of ploidy was four, and the highest level nine. Most individuals were hexaploid. Asexual reproduction was the most common mode of reproduction in all three species. Exclusively asexual populations had the broadest range of chromosome numbers and higher levels of ploidy (Fig. 3).

**Figure 4:**
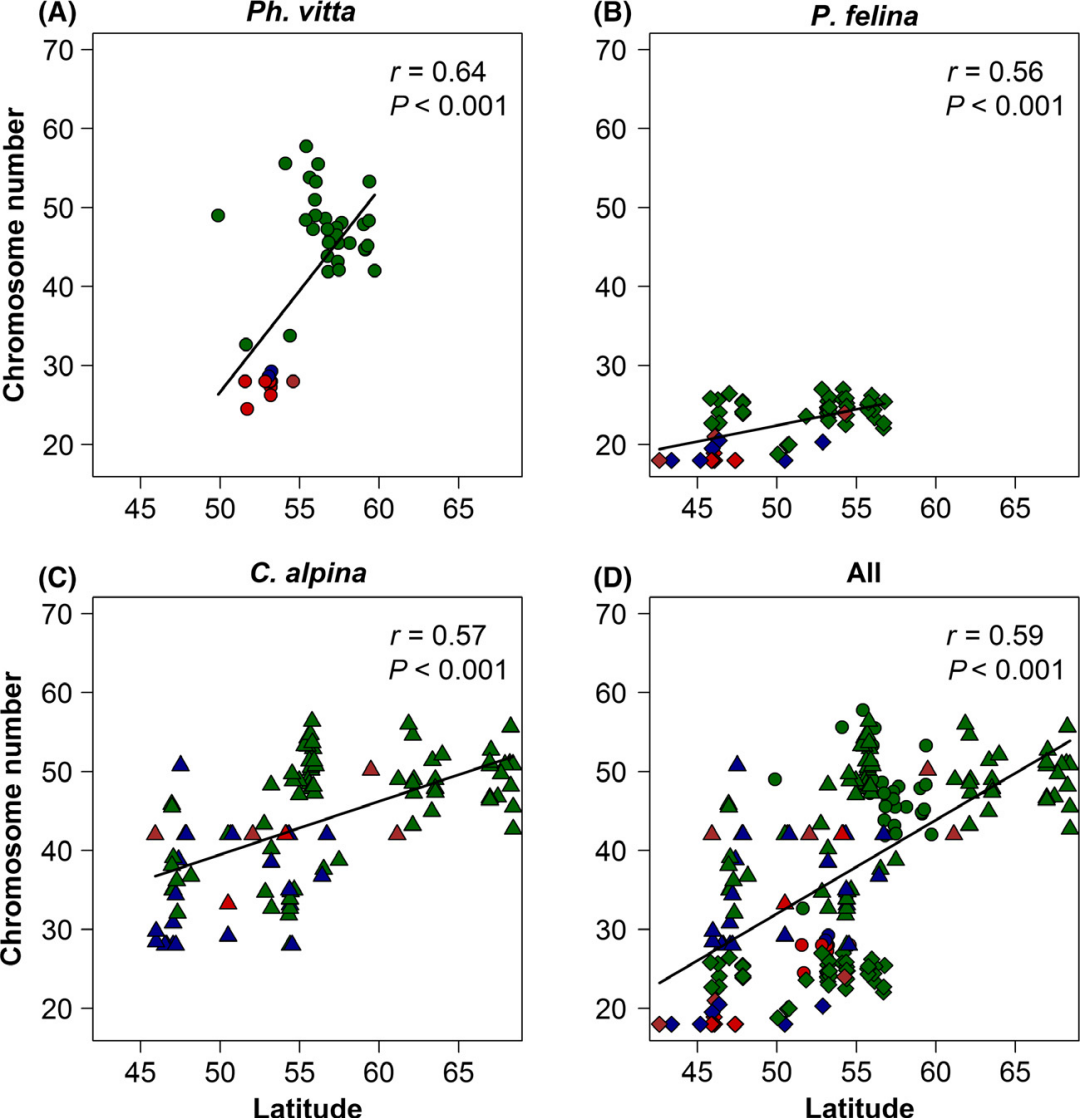
Relationship between latitude and mean chromosome numbers of the populations of three freshwater flatworm species (*Ph. vitta*,* P. felina* and *C. alpina*). (D) represents the relationship across all populations of all three species. Correlation coefficients and *P*‐values are from simple linear regressions. The different colors represent the reproduction mode of the populations: green = asexual, red = sexual, blue = sexual *and* asexual, brown = unknown.

A significant positive correlation between chromosome numbers and latitude was found for all three species (Fig. 4). Note that these relationships rest in general on the comparisons among the different reproduction modes within species (except the asexual populations of *C. alpina,* see Fig. 4). All environmental variables were significantly negatively correlated to latitude (see Appendix S1). Consequently across all populations of the three species, and with some exceptions also across populations within species, chromosome numbers decreased with increasing temperature, temperature range, precipitation, and NPP (see Appendix S4). The multiple linear model supported the effect of latitude and reproduction mode on chromosome number (Table 2). The significant species × reproduction interaction, however, indicated species‐specific differences in the strength of the relationships (Table 2). A more complex multiple linear model considering the principal components supported these findings (see Appendix S3).

**Table 2:** Statistics from a multiple linear model (Type I sum of squares) showing the effects of species, reproduction mode and latitude (explanatory variables) on the mean chromosome number (response variable) of flatworm populations (*n* = 220). For populations with unknown mode of reproduction, an additional factor level was created. Significant *P*‐values (*P *<* *0.05) are in bold

Chromosome number	df	SS	*F*	*P*
Species	2	17359	354	**<0.001**
Reproduction mode	3	4878	66.5	**<0.001**
Latitude	1	620.1	25.4	**<0.001**
Species × reproduction mode	6	1620	11.0	**<0.001**
Species × latitude	2	99.4	2.03	0.13
Reproduction mode × latitude	3	18.5	6.20	0.86
Residuals	202	4940		

In univariate analyses, the mean *within* individual CV in chromosome numbers of the populations increased with latitude for *C. alpina* (*P *=* *0.019) and *P. felina* (*P *=* *0.009). For *Ph. vitta*, no significant correlation was found (*P *=* *0.74; see Appendix S5). By contrast, we found no such relationship in the mean *among* individual CV in chromosome numbers of the populations and latitude. The multiple linear model, however, revealed that the reproduction mode was the only factor significantly related to both estimates of CVs in chromosome number (see Appendix S6, S7, S8).

## Discussion

The mean chromosome numbers of three species complexes of freshwater flatworms (*Ph. vitta*,* P. felina,* and *C. alpina*) increased with latitude as predicted from the first hypothesis (Fig. 4). It has been repeatedly suggested that for a colonization of extreme environments, rapid changes of the organization of the genome such as polyploidization are adaptive and more important than the slower processes of mutation and recombination (Stebbins 1971). Polyploidization may increase fitness due to higher genetic flexibility, pre‐adaptation, neo‐functionalization, and sophisticated modifications in epigenetic regulation of gene expression (Bierzychudek 1985; Soltis and Soltis 2000; Comai 2005; te Beest et al. 2011). A number of mechanistic explanations for this increased fitness have been suggested, such as increased heterozygosity, higher selfing rates (Soltis and Soltis 2000), gene dosage effects (Durka 2002), heterosis (Grant 1971), bigger cells (Stebbins 1971; Comai 2005), a higher intrinsic growth rate, and a higher rate of gene expression (Stebbins 1971; D'Souza et al. 2005). The increased fitness also facilitates invasion of new habitats (Kearney 2005) and thereby causes range expansions (Hijmans et al. 2007). However, polyploidy is also associated with handicaps. This includes slower cell division (Stebbins 1971), gene silencing, a potentially unbalanced gene expression, and a loss of sexual reproduction and therefore also recombination and DNA repair during meiosis (Comai 2005).

Our analyses of data of the three species groups of flatworms revealed a high variability of chromosome numbers across Europe. The levels of ploidy varied from diploid to decaploid (Fig. 2). This provides the possibility of recombination *via* sexual reproduction in southern regions as well as radical rearrangements of whole chromosome sets *via* asexual reproduction in northern habitats. Our above arguments rest on the assumption that in extreme and more variable environments fast changes in the genetic makeup are advantageous. However, such rapid changes may also be beneficial in more stable environments where biotic interactions due to an increasing number of competitors/antagonists as well as co‐evolutionary processes between host and parasites (Red Queen hypothesis, Van Valen 1973) are more important than adaptations to the environment.

Although the increase of chromosome numbers with latitude found in this study is consistent with the prediction of our first hypothesis, the question arises: How general are our findings? For example, flatworm species of the genus *Dugesia* that are predominantly distributed in the Mediterranean seem to contradict this relationship (Lázaro et al. 2009). While in Central Europe, only sexual reproducing diploids of the species *D. gonocephala* are known, both diploid sexual and triploid asexual *Dugesia* species are found in the Mediterranean. The populations of the *D. gonocepha* “group” analyzed by Dahm (1958) support these findings. As noted in the introduction, geographical analyses of chromosome numbers often make the implicit assumption that northern latitudes provide extreme environments for species. Yet in some cases, the opposite may be true. High temperatures and repeated desiccation of freshwater habitats in southern Europe may be an extreme environment for a species adapted to more northern habitats. For such species, the same arguments used for explaining the advantageousness of high chromosome numbers for species colonizing northern areas might apply for species colonizing southern regions. Obviously, the definition of an extreme environment depends on the biogeographic origin of the considered species.

Our results lend some support to the second hypothesis on the more frequent occurrence of asexual individuals at higher latitudes and on the interdependency between the mode of reproduction and the level of ploidy (Fig. 3). Populations with high chromosome numbers tended to reproduce asexually, whereas those possessing comparatively low and even ploidy levels (di‐, tetra‐, or hexaploid) reproduced sexually. Thus, geographic parthenogenesis also contributes to the geographic variation in chromosome number. One explanation for this phenomenon is that asexual populations tend to be favored in marginal environments with fewer biotic interactions (Cuellar 1977; Glesener and Tilman 1978; Bierzychudek 1985; Hörandl 2006). The habitats of the analyzed flatworm species – resource‐poor and isolated parts of rivers – support this assumption. Disadvantages of apomixis may be attenuated to a certain degree by higher levels of polyploidization. This is in accordance with the idea that polyploidy is advantageous for stable parthenogenesis (Suomalainen et al. 1987) and that an elevated degree of allopolyploidy rather than asexuality explains the wider geographic distribution of parthenogenetic species (Adolfsson et al. 2010). Polyploids are sometimes considered as fit (Comai 2005) and generalist pioneers (Salemaa 1984) with “general purpose genotypes” (Stebbins 1971), while parthenogens are often seen as relatively common and successful in general (Suomalainen et al. 1987; Turgeon and Hebert 1994). Faster colonization of new habitats results in higher initial frequencies of apomicts and inhibits the establishment of diploid sexuals (Hörandl 2006), which is particularly true for previously glaciated areas (Stebbins 1985). This tendency might explain the large geographical range and the relatively frequent occurrence of polyploid asexual populations of flatworms.

The third hypothesis concerning an increase of the variation of chromosome number within individuals with latitude was confirmed at least partly for two of the three species (see Appendix S5). Habitat disturbance, nutritional stress, and temperature‐related fluctuations might all lead to errors during cell division. Environmental conditions at high latitudes (and elevations) may therefore repeatedly induce the formation of polyploids (Ramsey and Schemske 1998; Hijmans et al. 2007; te Beest et al. 2011). Furthermore, neopolyploids suffer from high genomic instabilities (Comai 2005; D'Souza et al. 2005); thus, diploidization and genome downsizing enhance stability and reduce biochemical costs (Leitch and Bennett 2004; Comai 2005; Mráz et al. 2009). As a result of the elimination of single chromosomes, the frequency of aneuploidy and of the CV of a population increases. This suggests that the amount of individuals varying in chromosome number should increase with latitude where resources for the maintenance of multiple chromosome copies are particularly limited (Leitch and Bennett 2004). Moreover, asexual individuals mutate more quickly (Normark 1996; Sunnucks et al. 1997) which also promotes a higher CV in northern regions. These patterns – increased chromosome numbers, asexuality, aneuploidy and strong variation within a population – seem to be characteristic for northern populations.

## Conclusion

For a long‐lasting success in competition with their diploid progenitors, polyploids (i) have to enter a different ecological niche, (ii) have to acquire some advantages in reproductive output as well as colonization abilities, and (iii) should possess sufficient genetic variation for future evolutionary adaptations (Salemaa 1984; Durka 2002; Hörandl 2006; te Beest et al. 2011). Correlations of polyploidy and various factors such as climate, life form, breeding system, and hybridity suggest that multiple processes explain the success of polyploid species (Grant 1971; Hörandl 2006). Our study provides evidence for an increase of the ploidy level with latitude while polyploidization constitutes the background for physiological and ecological adaptations. Furthermore, high chromosome numbers may be stabilized by an asexual mode of reproduction. Polyploidization and asexual reproduction facilitate the colonization of previously glaciated areas, and hence, the current spatial distribution of polyploid species seems to be a consequence of the biogeographic history.

## Conflict of Interest

None declared.

## Supporting information


**Appendix S1.** Correlation matrix among chromosome numbers of three freshwater flatworm species (*Ph. vitta*,* P. felina* and *C. alpina*) and latitude with selected environmental variables and interrelationships (Pearson's product–moment correlation *r*). Significant values (where *P *<* *0.05) are in bold.
**Appendix S2**. (a) Loadings of environmental variables and latitude on principal components extracted from the correlation matrix. (b) SD and % of explained variance of each principal component.
**Appendix S3.** Statistics from a multiple linear model (Type I sum of squares) showing the effects of species, reproduction mode, PC1 and PC2 calculated among latitude and environmental variables (see methods) on the chromosome numbers (response variable) of flatworm populations (*n* = 220). For populations with unknown mode of reproduction an additional factor level was created. Significant *P*‐values (*P *<* *0.05) are in bold.
**Appendix S4.** Relationship between the mean chromosome numbers of populations of three species of Platyhelminthes (*Ph. vitta*,* P. felina* and *C. alpina*) and a selection of environmental variables. The lines represent linear regressions among populations within individual species. Here only significant regression lines (*P *<* *0.05) are shown. Note that across species all linear regressions (lines not shown) revealed significantly negative relationships (*P *<* *0.05).
**Appendix S5.** Relationship between latitude and the coefficient of variation (CV) in chromosome number of each population of three freshwater flatworm species (*Ph. vitta*,* P. felina* and *C. alpina*). Note that CVs represent population means of CVs within individuals; (d) represents the relationship across all populations of the three species. Correlation coefficients and the *P*‐values represent simple linear relationships. The different colors represent the reproduction mode of the populations: green = asexual, red = sexual, blue = sexual *and* asexual.
**Appendix S6.** Statistics from a multiple linear model (Type I sum of squares) showing the effects of species, reproduction mode and latitude (explanatory variables) on the CV in chromosome numbers (response variable) of flatworm populations (*n* = 203) *within* individuals. For populations with unknown mode of reproduction, an additional factor level was created. Significant *P*‐values (*P *<* *0.05) are in bold.
**Appendix S7.** Statistics from a multiple linear model (Type I sum of squares) showing the effects of species, reproduction mode and latitude (explanatory variables) on the CV in chromosome numbers (response variable) of flatworm populations (*n* = 205) *among* individuals. For populations with unknown mode of reproduction, an additional factor level was created. Significant *P*‐values (*P *<* *0.05) are in bold.
**Appendix S8.** Left: Mean variation of the coefficient of variation (CV) calculated *within* individuals for three species of flatworms. Right: Mean variation of the coefficient of variation (CV) calculated *among* individuals within populations for three species of flatworms. The different colors represent the different reproduction modes: green = asexual, red = sexual, blue = sexual *and* asexual. Bars represent means; error bars show ±SD.

**Table S1.** Data used to analyse the variation in chromosome numbers of flatworm populations: The rows represent the populations and the columns the geographic location (longitude, latitude), the reproduction mode, mean chromosome number and the considered environmental variables (Annual_Mean_Temperature_[°C], Mean_diurnal_range_[C°], Annual Precipitation [mm], NPP [gDM/m^2^/year]).

**Table S2.** Data used to analyse the coefficient of variation in chromosome numbers calculated *within* individuals (CV). For other variables see Table S1.

**Table S3.** Data used to analyse the coefficient of variation in chromosome numbers *among* individuals within populations (CV_population). For other variables see Table S1.
