# Supplementary material for: Chromosome numbers in three species groups of freshwater flatworms increase with increasing latitude
Source: Ecol Evol. 2016 Feb 3;6(5):1420–9. doi: 10.1002/ece3.1969 (PMC4775536; doi:10.1002/ece3.1969)
Supplement: Supplementary file 1 — Appendix S1. Correlation matrix among chromosome numbers of three freshwater flatworm species (Ph. vitta, P. felina and C. alpina) and latitude with selected environmental variables and interrelationships (Pearson's product–moment correlation r). Significant values (where P < 0.05) are in bold. Appendix S2. (a) Loadings of environmental variables and latitude on principal components extracted from the correlation matrix. (b) SD and % of explained variance of each principal component. Appendix S3. Statistics from a multiple linear model (Type I sum of squares) showing the effects of species, reproduction mode, PC1 and PC2 calculated among latitude and environmental variables (see methods) on the chromosome numbers (response variable) of flatworm populations (n = 220). For populations with unknown mode of reproduction an additional factor level was created. Significant P‐values (P < 0.05) are in bold. Appendix S4. Relationship between the mean chromosome numbers of populations of three species of Platyhelminthes (Ph. vitta, P. felina and C. alpina) and a selection of environmental variables. The lines represent linear regressions among populations within individual species. Here only significant regression lines (P < 0.05) are shown. Note that across species all linear regressions (lines not shown) revealed significantly negative relationships (P < 0.05). Appendix S5. Relationship between latitude and the coefficient of variation (CV) in chromosome number of each population of three freshwater flatworm species (Ph. vitta, P. felina and C. alpina). Note that CVs represent population means of CVs within individuals; (d) represents the relationship across all populations of the three species. Correlation coefficients and the P‐values represent simple linear relationships. The different colors represent the reproduction mode of the populations: green = asexual, red = sexual, blue = sexual and asexual. Appendix S6. Statistics from a multiple linear model (Type I sum of squ [file ECE3-6-1420-s001.docx]

**APPENDIX**

Appendix S1 Correlation matrix among chromosome numbers of three freshwater flatworm species (*Ph. vitta*, *P. felina* and *C. alpina*) and latitude with selected environmental variables and interrelationships (Pearson's product-moment correlation r). Significant values (where *P* < 0.05) are in bold.

|  | **Chromosomes** | **Latitude** | **Temperature** | **Temperature range** | **Precipitation** |
| --- | --- | --- | --- | --- | --- |
| **Chromosomes** |  |  |  |  |  |
| **Latitude** | **0.59** |  |  |  |  |
| **Temperature** | **-0.48** | **-0.73** |  |  |  |
| **Temperature range** | **-0.28** | **-0.45** | -0.02 |  |  |
| **Precipitation** | **-0.48** | **-0.42** | **0.28** | 0.05 |  |
| **NPP** | **-0.57** | **-0.64** | **0.82** | -0.08 | **0.49** |

Appendix S2 (a) Loadings of environmental variables and latitude on principal components extracted from the correlation matrix. (b) SD and % of explained variance of each principal component.

|  |  | PC1 | PC2 | PC3 | PC4 | PC5 |
| --- | --- | --- | --- | --- | --- | --- |
| a) | Latitude | -0.534 | 0.314 | -0.137 | 0.538 | -0.555 |
|  | Temperature | 0.529 | 0.192 | 0.406 | -0.198 | -0.692 |
|  | Temperature_range | 0.116 | -0.885 | 0.012 | 0.370 | -0.256 |
|  | Precipitation | 0.363 | 0.049 | -0.901 | -0.111 | -0.206 |
|  | NPP | 0.539 | 0.280 | 0.069 | 0.722 | 0.324 |
| b) | Standard deviation | 1.659 | 1.090 | 0.880 | 0.423 | 0.324 |
|  | % of explained variance | 0.551 | 0.238 | 0.155 | 0.036 | 0.021 |

**Appendix S3** Statistics from a multiple linear model (Type I sum of squares) showing the effects of species, reproduction mode, PC1 and PC2 calculated among latitude and environmental variables (see methods) on the chromosome numbers (response variable) of flatworm populations (n = 220). For populations with unknown mode of reproduction, an additional factor level was created. Significant *P*-values (*P* < 0.05) are in bold.

| Chromosome Number | Df | SS | *F* | *P* |
| --- | --- | --- | --- | --- |
| Species | 2 | 17359 | 405 | **< 0.001** |
| Reproduction mode | 3 | 4878 | 75.9 | **< 0.001** |
| PC1 | 1 | 1000 | 46.7 | **< 0.001** |
| PC2 | 1 | 6.1 | 0.284 | 0.59 |
| Species*reproduction mode | 6 | 1578 | 12.3 | **< 0.001** |
| Species*PC1 | 2 | 117.4 | 2.74 | 0.07 |
| Reproduction*PC1 | 3 | 49.2 | 0.765 | 0.52 |
| Species*PC2 | 2 | 118.1 | 2.76 | 0.07 |
| Reproduction*PC2 | 3 | 227.3 | 3.53 | **0.02** |
| Residuals | 196 | 4200 |  |  |

**
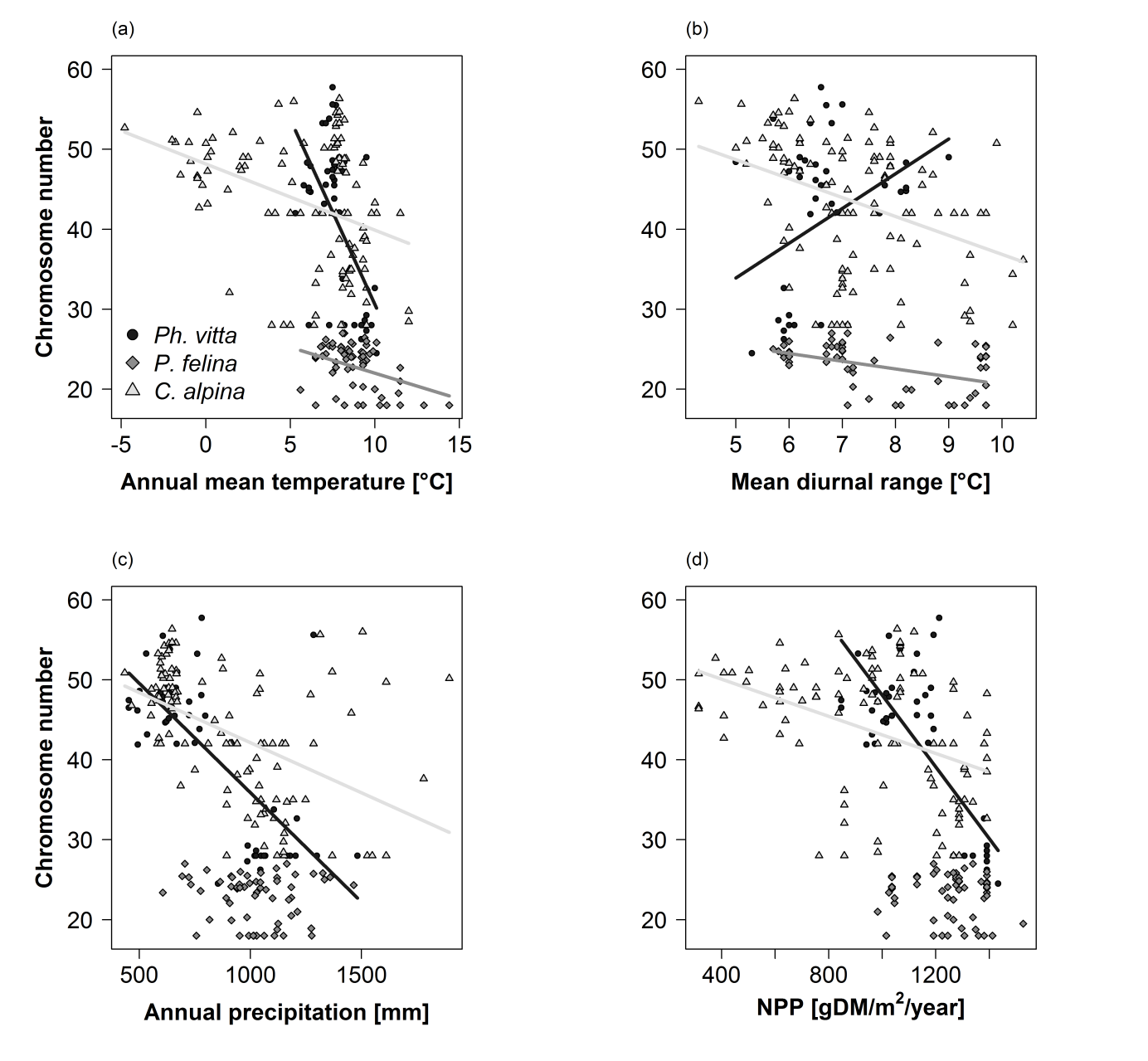
**

**Appendix S4** Relationship between the mean chromosome numbers of populations of the three species of Platyhelminthes (*Ph. vitta*, *P. felina* and *C. alpina*) and a selection of environmental variables. The lines represent linear regressions among populations within individual species. Here only significant regression lines (*P* < 0.05) are shown. Note that across species all linear regressions (lines not shown) revealed significantly negative relationships (*P* < 0.05).


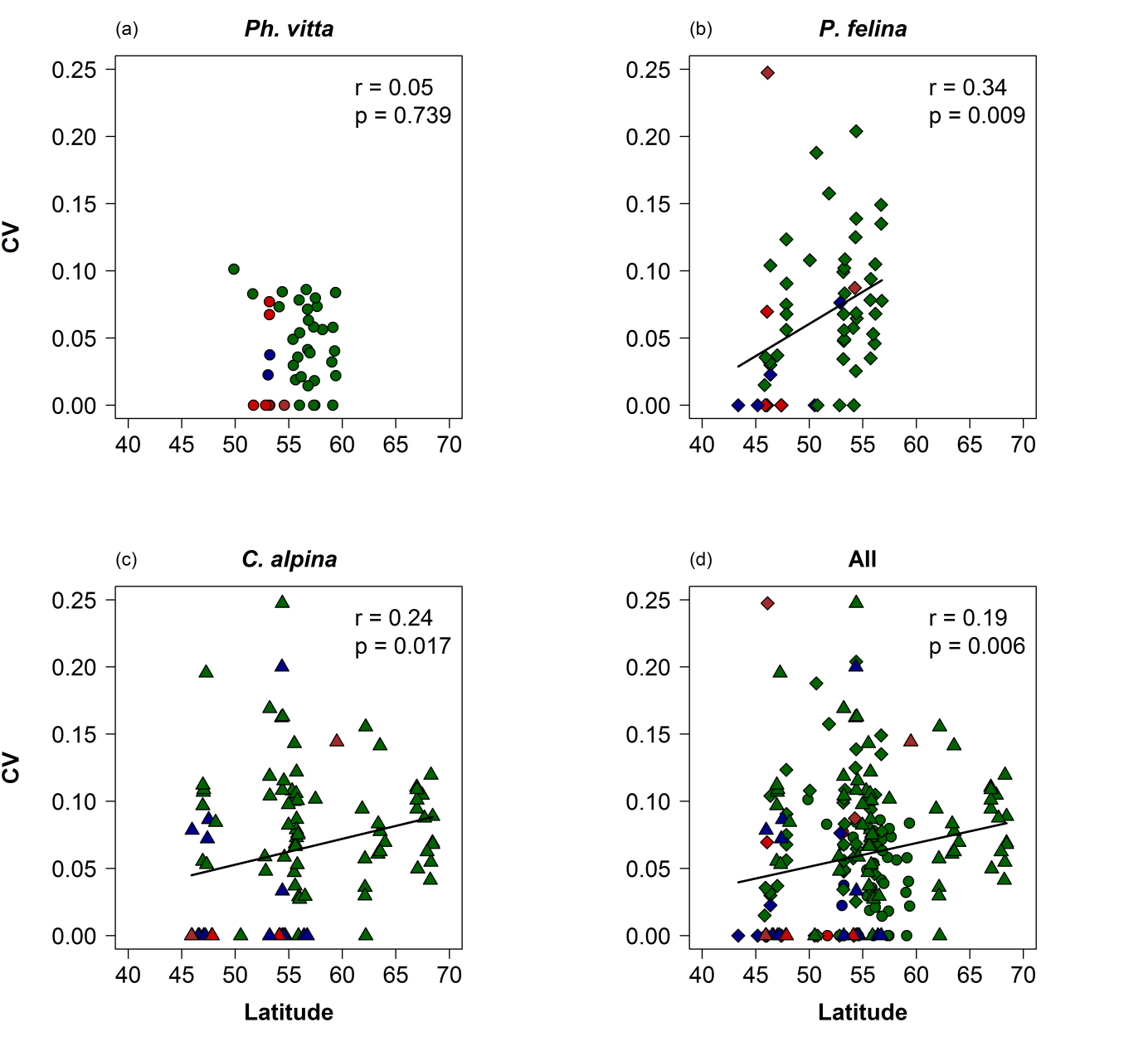


Appendix S5 Relationship between latitude and the coefficient of variation (CV) in chromosome number of each population of three freshwater flatworm species (*Ph. vitta*, *P. felina* and *C. alpina*). Note that CVs represent population means of CVs *within* individuals; (d) represents the relationship across all populations of the three species. Correlation coefficients and the *P*-values represent simple linear relationships. The different colours represent the reproduction mode of the populations: green = asexual, red = sexual, blue = sexual *and* asexual.

**Appendix S6** Statistics from a multiple linear model (Type I sum of squares) showing the effects of species, reproduction mode and latitude (explanatory variables) on the CV in chromosome numbers (response variable) of flatworm populations (n = 203) *within* individuals. For populations with unknown mode of reproduction, an additional factor level was created. Significant *P*-values (*P* < 0.05) are in bold.

| CV | Df | SS | *F* | *P* |
| --- | --- | --- | --- | --- |
| Species | 2 | 0.022 | 5.38 | **0.005** |
| Reproduction mode | 3 | 0.117 | 18.8 | **<0.001** |
| Latitude | 1 | <0.001 | 0.19 | 0.66 |
| Species*reproduction mode | 6 | 0.017 | 1.35 | 0.24 |
| Species*latitude | 2 | 0.007 | 1.71 | 0.18 |
| Reproduction mode*latitude | 3 | 0.003 | 0.48 | 0.7 |
| Residuals | 185 | 0.384 |  |  |

**Appendix S7** Statistics from a multiple linear model (Type I sum of squares) showing the effects of species, reproduction mode and latitude (explanatory variables) on the CV in chromosome numbers (response variable) of flatworm populations (n = 205) *among* individuals. For populations with unknown mode of reproduction, an additional factor level was created. Significant *P*-values (*P* < 0.05) are in bold.

| CV Population | Df | SS | *F* | *P* |
| --- | --- | --- | --- | --- |
| Species | 2 | 0.001 | 0.15 | 0.87 |
| Reproduction mode | 3 | 0.081 | 9.85 | **<0.001** |
| Latitude | 1 | 0.001 | 0.23 | 0.63 |
| Species*reproduction mode | 5 | 0.008 | 0.6 | 0.7 |
| Species*latitude | 2 | <0.001 | 0.02 | 0.98 |
| Reproduction mode*latitude | 3 | 0.002 | 0.26 | 0.86 |
| Residuals | 188 | 0.513 |  |  |


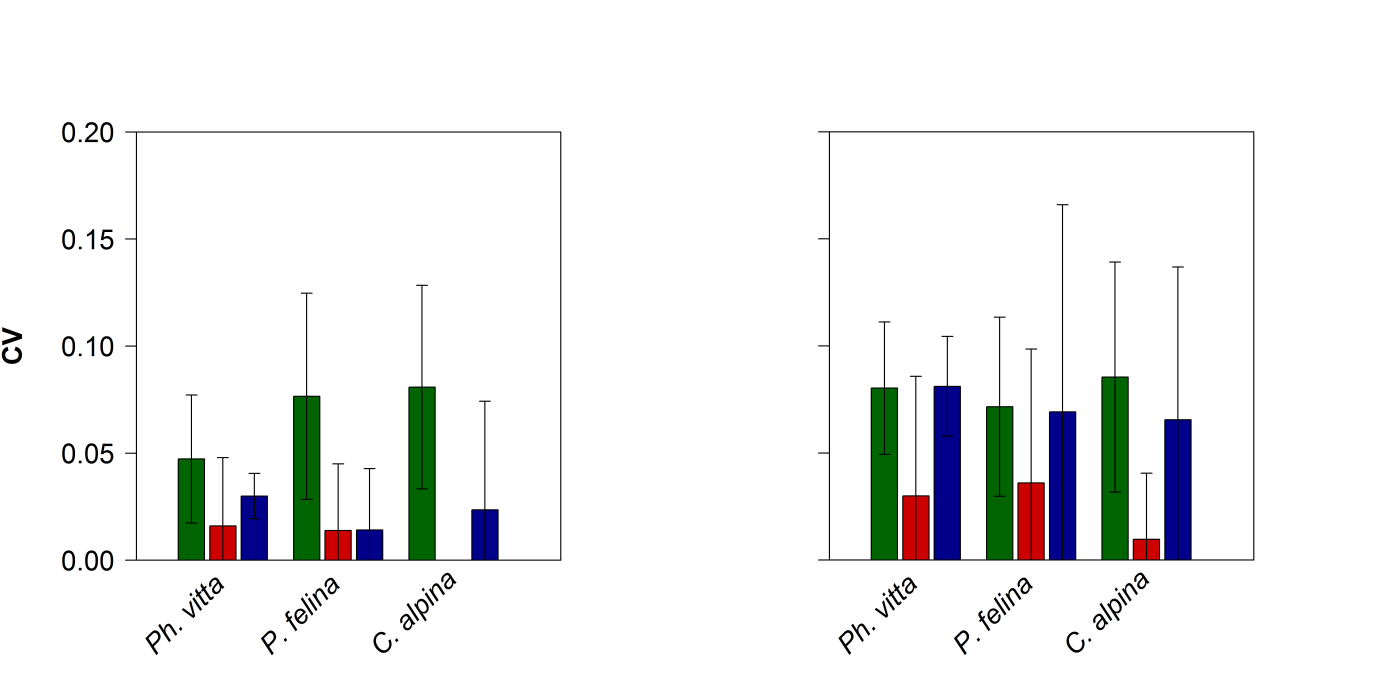


Appendix S8 Left: Mean variation of the coefficient of variation (CV) calculated *within* individuals for three species of flatworms. Right: Mean variation of the coefficient of variation (CV) calculated *among* individuals within populations for three species of flatworms. The different colours represent the different reproduction modes: green = asexual, red = sexual, blue = sexual *and* asexual. Bars represent means; error bars show ± SD.
